# A New Species of *Bryaxis* (Coleoptera: Staphylinidae: Pselaphinae) from Mount Etna (Sicily, Italy) and Notes on Its Ecology and Distribution

**DOI:** 10.3390/ani13182941

**Published:** 2023-09-16

**Authors:** Giorgio Sabella, Giuseppe Nicolosi

**Affiliations:** 1 Department of Biological, Geological and Environmental Sciences, University of Catania, Via Androne 81, 95124 Catania, Italy; 2 Department of Life Sciences and Systems Biology, University of Turin, Via Accademia Albertina 13, 10123 Torino, Italy; giuseppe.nicolosi@unito.it

**Keywords:** Pselaphinae, *Bryaxis*, Mount Etna, lava caves, Sicily

## Abstract

**Simple Summary:**

A new species, *Bryaxis aetnensis* sp. nov., has been found on Mount Etna in Sicily, Italy. The species is closely related to the *Bryaxis difficilis* group, a set of similar beetles found in Sicily and Sardinia. The study describes the unique features and distribution of the Sicilian beetles in this group. *Bryaxis aetnensis* resembles another species, *B. marinae*, but can be distinguished by its darker color, longer antennal scape and terminal palpomere, and distinct reproductive organ structure. *Bryaxis aetnensis* has been discovered at various altitudes on Mount Etna, indicating its adaptability to different environments. This adaptability might explain the observed morphological variability among individuals from different sampling locations. This discovery emphasizes the importance of further exploration on Mount Etna, and why protecting these newly explored areas is crucial. As Mount Etna is relatively young in terms of its volcanic activity, studying the recent colonization of species in this environment provides valuable insights.

**Abstract:**

A new species of the subfamily Pselaphinae (Coleoptera: Staphylinidae) has been discovered on Mount Etna (Sicily, Italy) and is described herein as *Bryaxis aetnensis* sp. nov. The new species is closely associated with the *Bryaxis difficilis* group, a highly homogeneous group of species living in the regions of Sicily and Sardinia. Diagnostic features and distribution of Sicilian species of this group are treated and illustrated herein. *Bryaxis aetnensis* sp. nov. exhibits similarities to *B. marinae* but can be distinguished by the darker color, longer antennal scape and terminal palpomere, and in the aedeagus morphology. The distribution of *B. aetnensis* sp. nov. spans a wide altitudinal range, demonstrating a remarkable climatic tolerance across the slopes and diverse habitats of Mount Etna. This broad tolerance reflects the species’ probable high ecological plasticity, which may also contribute to the observed morphological variability among individuals from different sampling sites. The significance of this new discovery on Mount Etna highlights the need to intensify sampling efforts in the region. Strengthening protection for these unexplored environments is crucial, and it also aids in unraveling biogeographic questions about the fauna inhabiting the area. As a relatively young volcanic environment, species colonization has occurred recently, making it an intriguing subject of investigation.

## 1. Introduction

The genus *Bryaxis* Kugelann, 1794, is one of the largest pselaphine genera, comprising 385 species and 40 subspecies [1,2]. Its distribution primarily spans the Palaearctic Region, showcasing a wide array of remarkable species. There have been sporadic reports suggesting its presence in the Oriental Region, specifically in Myanmar and Laos, although these claims require further confirmation as they are based solely on female specimens [3]. As such, the taxonomy and biogeography of *Bryaxis* remain intriguing subjects of study, prompting the need for more comprehensive research and exploration across various geographical regions.

Until now, 82 species and 13 subspecies of *Bryaxis* have been documented in Italy [4]. Currently, Sicily presents a total of seven known species of this genus. Remarkably, four of these species are endemic to the island: *B. siculus* (Fiori, 1913), *B. nebrodensis* Besuchet, 1980, *B. marinae* Sabella, 1989, and *B. degiovannii* Poggi & Colacurcio, 2021 [5,6,7,8]. They belong to a highly homogeneous group known as the *Bryaxis difficilis* group, characterized by possessing aedeagi of a primitive type, featuring simple parameres, and limited differentiation in the armature of the internal sac. Furthermore, males within this group lack secondary sexual characters on both the first and second segments of their antennae [7].

The remaining three Sicilian species exhibit a wider distribution. *Bryaxis curtisii curtisii* (Leach, 1817) can be found in multiple countries, including Austria, Belgium, the Czech Republic, Denmark, France, Germany, Ireland, Italy, Liechtenstein, the Netherlands, Sweden, Switzerland, and the United Kingdom [9,10,11]. *Bryaxis italicus* (Baudi di Selve, 1870) is widely distributed in Sicily but also across the Italian Peninsula, Canton of Ticino (Switzerland), and Southern France, particularly the Var and Alpes-Maritimes regions. Lastly, *B. picteti meridionalis* (Machulka, 1932) is found in the Southern Apennines and Sicily [9,10,11].

Although systematic investigations have been carried out by zoologists from the University of Catania (e.g., Caruso [12]), our understanding of volcanic subterranean environments remains limited, particularly in the field of biospeleology. Furthermore, the presence of a *Bryaxis* species has not been reported in previous studies conducted in lava caves.

The faunistic study of environments deserving protection, such as lava caves, utilizing sampling methods commonly considered invasive, like pitfall traps, is motivated by the necessity to enhance our understanding of the faunal populations inhabiting the volcanic caves of Mount Etna. Furthermore, the pitfall trap method, when employed professionally, does not cause declines in subterranean populations. As previous studies have highlighted, caves represent only a small fraction of the overall habitat available to these species. Hypogean arthropods primarily inhabit the narrow fissures of rocks, rather than the accessible subterranean spaces between rocks, such as caves (for a comprehensive review on the topic, see Mammola et al. [13]).

The use of extensive field searches using this methodology in the lava caves of Mount Etna allowed the discovery of a new species of *Bryaxis,* which is described here. Detailed illustrations of major diagnostic features are provided, encompassing observations from male and female specimens. Additionally, a thorough comparative analysis of body traits was undertaken, shedding light on the species’ variations in relation to altimetric range.

## 2. Materials and Methods

### 2.1. Study Area

Sampling activities were carried out on Mount Etna (Sicily, S-Italy), a quaternary stratovolcano situated in northeastern Sicily. It reaches an altitude exceeding 3330 m [7], standing as the highest peak in Sicily. The volcano has a total area of approximately 1250 km^2^, with a perimeter of over 135 km, delimited by the Peloritani mountains to the north, Nebrodi to the northwest, and Simeto river and the alluvial plain (“Piana di Catania”) to the south and southeast. This remarkable area has been protected since 1987 through the establishment of the “Parco dell’Etna” (EUAP0227) and was further recognized as a UNESCO Heritage site in 2013 due to its outstanding biodiversity and unique geological features. The volcanic substrates present various ages, ranging from the very recent 2014/2015 lava flow to approximately 500,000-year-old tholeitic basalts found in a small area along the Ionian coast of Catania [14]. Due to the high frequency of volcanic activity and the opening of eruptive fissures on its flanks, the slopes of Mount Etna have undergone continuous modifications, particularly in the summit areas [15]. These distinctive environmental conditions on Mount Etna have fostered rich biological diversity (e.g., Sciandrello et al. [16]; Caruso [17]; Magrini et al. [18], Nicolosi et al. [19,20]), leading to its recognition as a habitat of great significance. The vegetation is frequently affected by volcanic activities, causing dramatic changes in the structure, density, cover, floristic composition, species richness, and diversity [21].

Mount Etna features over 200 basaltic lava tubes [22]. While the prevailing notion suggests that lava tubes predominantly form on *pahoehoe* lavas (smooth glassy basalt lava), the formation of caves on Mount Etna frequently occurs in *aa* lava (rough brecciated basalt lava), and certain tubes that are traditionally considered to have formed on *pahoehoe* lavas are found within the massive expanse of *aa* lava flows (e.g., Calvari [23]).

These tubes are distributed across different altitudes and volcanic substrates of varying ages, providing a diverse range of ecological and microclimatic conditions. The presence of such unique and varied habitats contributes to the extraordinary ecological richness and complexity found on Mount Etna, making it a captivating site for researchers to explore the interactions between volcanic processes and the living organisms that have adapted to thrive in this dynamic volcanic landscape.

### 2.2. Sampling Design

Field activities were conducted between the years 2020–2022 in 25 different caves, spanning altitudes ranging from 615 to 1718 m, as detailed in Nicolosi et al. [19]. The methodology involved the use of pitfall trapping consisting of one 500 mL stacked glass container, 10 cm high and 10 cm diameter. These traps were baited with meat and crushed bones. At each designed sampling site, two pitfall traps were positioned—one near the cave entrance and another in the innermost section. They were replaced at regular intervals of approximately every 6 months, from summer 2020 to winter 2022. All collected material was initially preserved in 70% ethanol, sorted in the laboratory, and subsequently stored in the G. Sabella collection and preserved in the section of Animal Biology of the Department of Biological Geological and Environmental Sciences of University of Catania (DBUC). Additionally, cave air temperature measurements were taken using iButton dataloggers at each sampling site. Lava ages were obtained from Etna Volcano’s Geological map (1:50,000 scale) [24].

## 3. Results

### 3.1. Taxonomy

Subfamily PSELAPHINAE Latreille, 1802

Genus *Bryaxis* Kugelann, 1794

#### *Bryaxis aetnensis* sp. nov. (Figure 1A)

zoobank.org:act:7FDBBD26-7549-4E56-9EC5-FE3CD1D053C8

Type material: Holotype: SICILY—Etna Mount: Catania province: Linguaglossa Municipality: Grotta dei Rotoli [SICT1239], 1.488 m, trap 2, 06.I.2022, G. Nicolosi leg., 1 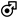 (DBUC); Paratypes (12 ex.): SICILY—Etna Mount: Catania province: Linguaglossa Municipality: Grotta di Monte Corruccio [SICT1056], 1.389 m, trap 2, 06.I.2022, G. Nicolosi leg., 1 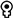 (DBUC); same data, trap 2, 22.VII.2022, G. Nicolosi leg., 1 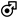 (DBUC); Grotta del Porcospino [SICT1033], 982 m, trap 2, 28.VII.2021, G. Nicolosi leg., 1 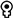 (DBUC); same data, trap 2, 26.XII.2021, G. Nicolosi leg., 1 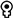 (DBUC); same data, trap 2, 13.VIII.2022, G. Nicolosi leg., 1 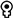 (DBUC); Mascali Municipality: Grotta Forcato [SICT1013], 670 m, traps, 01.VIII.2021, G. Nicolosi leg., 1 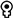 (DBUC); Trecastagni Municipality: Grotta di Monte Cicirello [SICT1156], 990 m, traps, 29.XII.2020, G. Nicolosi leg., 1 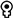 (DBUC); Nicolosi Municipality: Grotta Lunga (di Monpeloso) [SICT1029], 820 m, 21.VII.2022, G. Nicolosi leg., 2 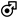

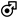
 (DBUC); same location, trap 2, 26.XII.2021, G. Nicolosi leg., 1 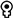 (DBUC); Adrano Municipality: Grotta del Santo (di San Nicola) [SICT1032], 1.041 m, trap. 2, 06.I.2022, G. Nicolosi leg., 1 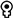 (DBUC); same location; trap 2, 02.VIII.2022, G. Nicolosi leg., 1 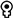 (DBUC).

**Diagnosis:** Body length: 1.45–1.75 mm; dorsal surface covered with sparse and decumbent pubescence. Eyes with 2–4 ommatidia. ***Male***. Antennomeres: 1 and 2 unmodified, similar to those of the female; protibiae: very barely incised in the distal quarter; metatibiae: weakly incised and sinuate in the distal quarter. Aedeagus with a short and ovoid basal capsule, and stout and subparallel parameres, widened in the basal third, and sinuated and narrowed in the apical portion; the internal sac with two apical pieces.

**Figure 1:**
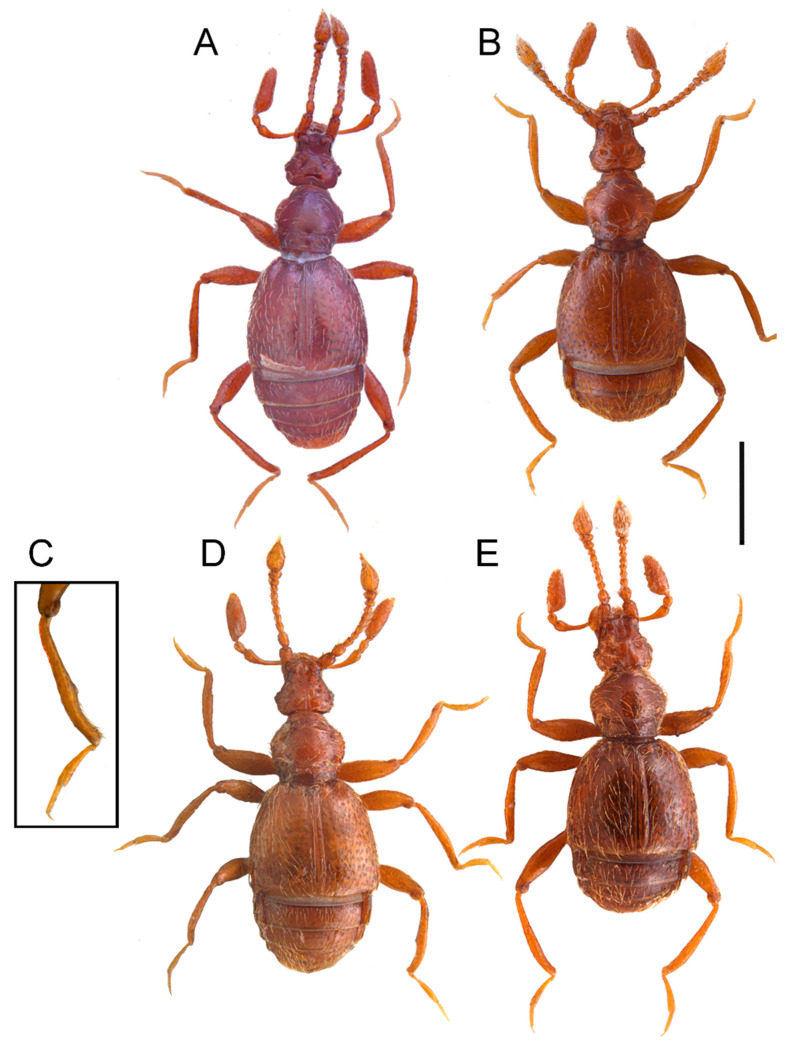
Dorsal habitus of *Bryaxis* species. *B. aetnensis* sp. nov. (**A**). *B. marinae* (**B**). *B. nebrodensis* (homomorphous male) (**C**). *B. nebrodensis* (oedymerous male) (**D**). *B. siculus* (**E**). Scale bar: 1 mm.

**Description**: Body length: 1.45–1.75 mm, entirely testaceous reddish or reddish, wingless (Figure 1A). Pubescence decumbent, consisting of setae (length: 0.05–0.06 mm), sparse over the entire body and by shorter and suberect yellowish bristles, which are particularly dense on antennae and palpomere 4.

Head slightly wider (0.27–0.29 mm) than long (0.265–0.275 mm), distinctly narrower than the pronotum. Frontal lobe 0.19 mm wide. Frons with an inverted U-shaped deep median depression, its surface very finely punctate behind the antennal tubercles, the latter raised. Vertex crossed by a median longitudinal carina reaching up to the anterior edge of vertexal foveae, the latter evident and situated anteriorly at the level of anterior eye margin. In the lateral view, an evident ocular-mandibular carina can be observed. Eyes with 2–4 ommatidia. Tempora rounded. Posterior region of gular region with some long seta. Median gular carina lacking.

Maxillary palpi with palpomere 2 gradually enlarged in the distal third; its surface with about 10 slightly raised tubercles; palpomere 3 just longer than wide, its surface with some tubercles; palpomere 4 about three times longer (0.275–0.285 mm) than wide (0.08–0.09 mm) with the greatest width in the basal third; its surface densely pubescent.

Antennae 0.62–0.68 mm long. Scape subcylindrical about twice as long as wide without modification. Pedicel distinctly longer than wide, and slightly narrower than the scape, without modifications. Antennomere 3 distinctly longer than wide, slightly narrowed at the base; antennomeres 4 and 5 slightly longer than wide; antennomeres 6 and 7 as long as wide; antennomere 8 just wider than long; antennomere 9 distinctly wider than the previous ones and shorter than 8; antennomere 10 wider than 9 and about one and a half times wider than long; antennomere 11 distinctly longer than wide (0.175/0.08 mm) and longer than the previous four considered together.

Pronotum wider than long or about as long (0.33–0.35 mm) as wide (0.34–0.35 mm), with maximum width approximately halfway along its length, anteriorly tapered with sinuates sides, posteriorly more lightly tapered with straight sides. Its dorsal surface shiny with some rare and faint punctures with an evident antebasal sulcus that connects the median antebasal fovea with the larger lateral antebasal foveae. The tegument between the pronotal posterior margin and the antebasal sulcus slightly rough. In the lateral view, an evident oblique pleural carina can be observed. Shiny metasternum with a slight median depression. Base of mesocoxal cavities with a pubescent pit on each side.

The combined width of elytra (0.60–0.63 mm) more than their length (0.56–0.58 mm), convex with sides slightly rounded from base to the apex, widest just before the apex. Humeral protuberance not well developed. Dorsal surface with some punctures. Each elytron with basal, discal, and subhumeral foveae. Sutural and marginal stria reaching about elytral apex, discal stria reaches about a half of the elytral length. Abdomen normally shaped without particular characters.

**Figure 2:**
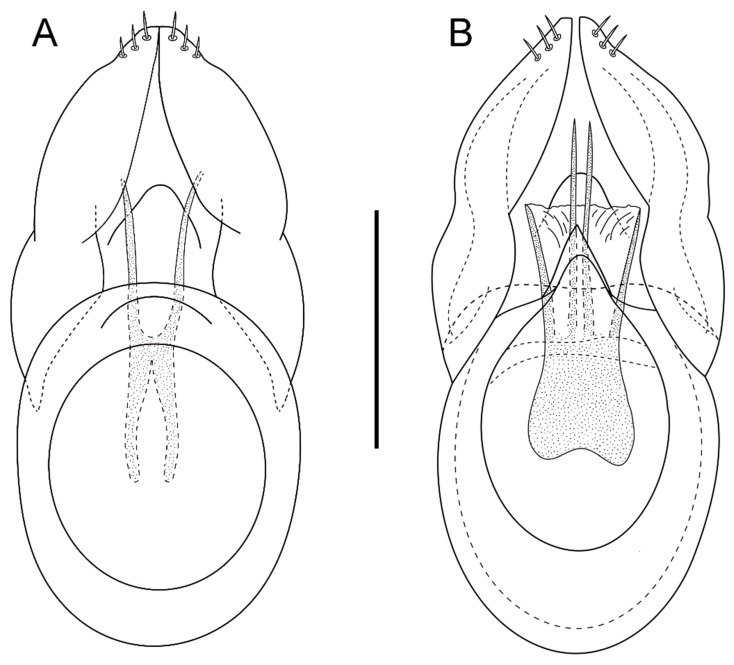
Aedeagus of *Bryaxis aetnensis* sp. nov. (**A**) and *Bryaxis marinae* (**B**) in dorsal view. Scale bar: 0.1 mm.

***Male***: Protibiae very barely incised in the distal quarter, metatibiae weakly incised and sinuate in the distal quarter. Gular region slightly swollen with transverse sulcus (depréssion gulaire according to Besuchet and Kurbatov [3], anteriorly limited by a slender carina. Aedeagus 0.26 mm long with short and ovoid basal capsule with a large dorsal window and stout and subparallel parameres, widened in the basal third, sinuated and narrowed in the apical portion; the internal sac with two apical pieces (Figure 2A).

***Female***: Very similar to male with the gular region and tibiae unmodified.

**Etymology:** Named after Mount Etna volcano, the sole location in Sicily where the species was collected.

### 3.2. Distribution and Ecology Notes

**Figure 3:**
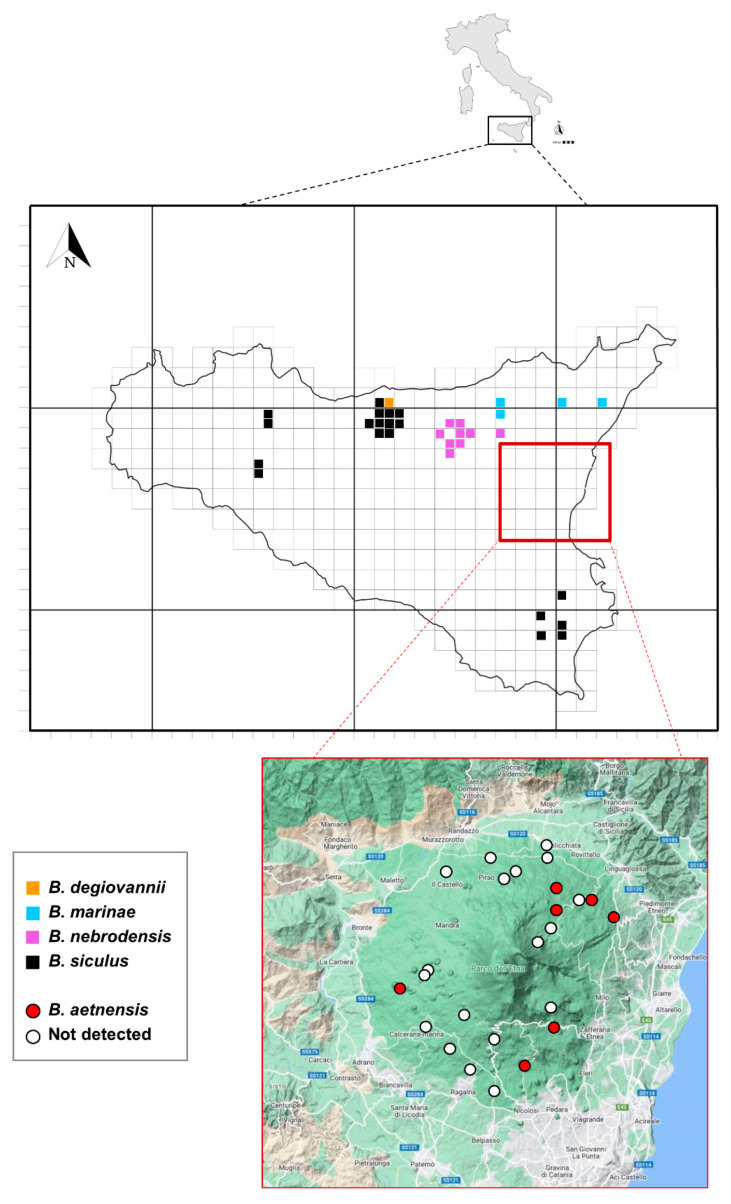
Distribution of species within the *Bryaxis difficilis* group in Sicily (**above**) and the newly described *Bryaxis aetnensis* sp. nov. on Mount Etna (**below**) (according to literature and unpublished data).

The distribution of species of the *Bryaxis difficilis* group in Sicily is depicted in Figure 3 (from Sabella [25], modified). *Bryaxis aetnensis* sp. nov. is currently known exclusively from the Mount Etna area, while *B. marinae* can be found in the Peloritani Mountains, and on the eastern and southern slopes of the Nebrodi Mountains. Interestingly, *B. marinae* appears to have a vicariant distribution, with the western and northern slopes inhabited by *B. nebrodensis*. *Bryaxis siculus* is present in the woods of northwestern Sicily and Monti Sicani and southeastern Sicily (Iblean district). Currently, *B. degiovannii* is only known from a single specimen collected in Isnello (Madonie). The field activities on Mount Etna led to the discovery of seven occurrences of *B. aetnensis* sp. nov. out of 25 sampled lava caves. Six of them are included within Mount Etna Natural Park and only “Grotta Forcato” falls outside the borders of the park.

Despite previous studies conducted on the lava caves of Mount Etna (e.g., Caruso [12]), this investigation presents the first documented occurrence of a new species to science of the *Bryaxis* genus on the volcano. The successful detection of this new species was primarily achieved through the meticulous use of pitfall traps. Although *B. aetnensis* sp. nov. is not strictly subterranean, all specimens were exclusively collected from the deeper sections of the caves using pitfall traps, with no findings near the cave entrance.

Beetles belonging to the genus *Bryaxis* are found in many different environments. Many are linked to litter or detritus, which is always humid, but some can also be gathered among mosses, in rotting mushrooms or wood, among the roots of riparian vegetation, and under well sunken stones.

A number of species of *Bryaxis* are considered troglobitic, meaning they are adapted to cave-dwelling environments (see, for example, Poggi [26] for Italian fauna) or more broadly to the Milieu Subterraine Superficiel (MSS). However, the presence of young substrates from the recent volcanic systems characterizing Mount Etna has led to a relatively impoverished soil fauna compared to other areas of Sicily. In fact, the volcano has a relatively young age, with its activity divided into four well-defined and spatially localized phases: Aci Trezza Synthem, Adrano Synthem, Timpe Supersynthem, and Valle del Bove Supersynthem. The oldest phases, Aci Trezza Synthem, were intruded in clay sediments or emplaced on the seafloor about 500,000 years ago. Subsequent subaerial products of the Adrano Synthem erupted approximately 330,000 years ago. The Timpe Supersynthem, starting from about 220,000 years ago, saw increased eruptive activity leading to the formation of a lava shield. The Valle del Bove Supersynthem, at least 110,000 years ago, exhibited central-type activity and gave rise to the earliest recognized volcanic centers. The Stratovolcano Supersynthem, from about 57,000 years ago, witnessed intense eruptive activity forming a high stratocone. Finally, the Mongibello volcano, built from effusive activity in the last 15,000 years, marked the culmination of Mount Etna’s volcanic history [14,24,27]. The unique combination of young substrates and frequent disturbances caused by volcanic activity in the area likely accounts for the notable presence of *Bryaxis* within the subterranean habitats of Mount Etna. These subterranean environments offer micro-climatic conditions that are more favorable for the species’ survival, providing stable temperatures and higher humidity levels.

### 3.3. Habitat Description and Body Size Variation

The species occurs along a relatively wide altitudinal range, from 670 (“Grotta Forcato”) to 1488 m a.s.l. (“Grotta dei Rotoli”) (mean ± SD = 1054 ± 292 m; N = 7). When considering cave temperatures, occurrence sites range from 6.49 (“Grotta di Monte Corruccio”) to 12.82 °C (“Grotta del Santo”) (average 10.48 ± 2.48 °C; N = 6). Moreover, the species is independently distributed with respect to substrate age, considering the ability to colonize caves that opened very recently (<500 years) and older (>1000 years) lava fields presenting different stages of vegetation colonization. Specimens were mostly collected in relatively recent lava caves, where the substrate is devoid of soil (“Grotta dei Rotoli”) or with thin soil cover (“Grotta Forcato” and “Grotta di Monte Corruccio”). These lava caves are most commonly dominated by the shrub *Genista aetnensis* (Biv.) DC., 1825, playing a crucial role in the colonization of Etna lavas [28]. In contrast, on older lava substrates where the soil has developed to a certain thickness (e.g., “Grotta di Monte Cicirello” and “Grotta del Santo”), the shrub communities include other woody species. These areas frequently harbor species from the herbaceous vegetation category, and species belonging to the woodland vegetation dominated by *Quercus* species, such as *Quercus ilex* L., 1753 and *Q. pubescens* Willd., 1805. Details on the sampled localities of *Bryaxis aetnensis* sp. nov. on Mount Etna are provided in Table 1.

An assessment of variable body traits in *B. aetnensis* sp. nov. highlights the substantial variation between sampling localities (Table S1).

**Figure 4:**
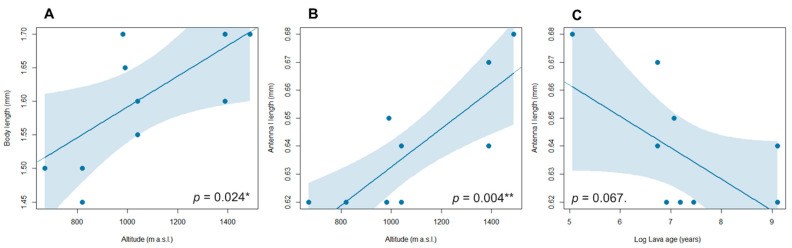
Predicted linear relationship (blue line) and 95% confidence interval (blue shading) between altitude and body traits (body and antenna I lengths) (**A**,**B**) and between lava age and antenna I length (**C**) in *Bryaxis aetnensis* sp. nov., derived from the linear model. Lava age is Log-transformed.

When examining the relationship between body size traits and biogeographic features (Figure 4), we discovered a significant positive relationship between altitude and body length (Est: 0.254; Std Err.: 0.094; *p*: 0.024*). A similar pattern was observed between altitude and antenna I elongation (Est: 0. 0.070; Std Err.: 0.019; *p*: 0.004**). Additionally, we found an inverse relationship between antenna I elongation and the age of the sampled lava cave (Est: −0.012; Std Err.: 0.005; P: 0.067.), although the relationship was only approaching statistical significance.

## 4. Discussion

Sicily represents a compelling area for study due to its complex geological composition, characterized by the simultaneous occurrence of carbonate, evaporite, and volcanic terrains. This unique geological complex has given rise to a remarkable diversity of surface formations and a significant number of hypogean environments [29]. Recent extensive monitoring activities undertaken in the past few years have shed light on the presence and abundance of various species within these subterranean habitats. One notable group of organisms that has garnered attention in these recent studies is the Pselaphinae, particularly with the discovery of several species inhabiting subterranean habitats (e.g., Sabella et al. [30,31,32]. Despite efforts to foster knowledge on the subterranean fauna living on Mount Etna (e.g., Caruso [12,17], D’Urso et al. [33]), it is only recently that more extensive sampling activities have brought attention to these largely unexplored habitats (e.g., Nicolosi et al. [19]). As a result, a new troglobitic species of the Pselaphinae genus *Briaxys* has been discovered and is described herein.

*B. aetnensis* sp. nov. belongs to the *B. difficilis* group as defined by Sabella [7]. This group has a Sardinian–Sicilian geonomy and includes, up to now, seven species, three from Sardinia: *Bryaxis difficilis* (Reitter, 1884); *B. odontogena* (Dodero, 1919); *B. subdentatus* (Dodero, 1919), and four from Sicily: *B. siculus* (Fiori, 1913) (Figure 1E), *B. nebrodensis* Besuchet, 1980 with homomorphous (Figure 1C) and oedymerous males (Figure 1D); *B. marinae* Sabella, 1989 (Figure 1B), and *B. degiovannii* Poggi & Colacurcio, 2021 [5,6,7,8,34,35,36]. The species belonging to this group, with evident Sardinian–Sicilian geonemia, are of notable biogeographical interest, being presumably ascribable to the ancient elements of the Sicilian and Sardinian fauna. Their origin could be traced back to the pre-Pliocene era, making them paleoendemics. The current distribution of these species can be explained by considering the complex geological history of the Mediterranean, which is in line with the most recent views related to the theory of microplate migration in the western Mediterranean starting from the Oligocene [37,38,39], (see also Sabella [25] for further discussions on this topic).

The Sicilian species (Figure 1) are all distinguished from the Sardinian species by an evident microphthalmia (eyes formed by 1–6 ommatidia in Sicilian species vs. eyes with more than 10 ommatidia in Sardinian species). They are otherwise very similar to each other and differ only in some features, such as the length of the antennal scape and of the last palpomere, and also in the secondary sexual characteristics of the males (morphology of the pro- and metabiae and of the aedeagus).

The Sicilian species *B. aetnensis* sp. nov. seems more similar to *B. marinae*, from which it is distinguished by the darker color, the longer antennal scape (0.11–0.12 mm against 0.09–0.1 mm), the longer last palpomere (0.275–0.285 mm against 0.245–0.26 mm), and the morphology of the aedeagus (cfr. Figure 2A,B).

*B. aetnensis* demonstrates a wide climatic tolerance across all slope of the volcano, ranging from 670 up to 1488 m. Furthermore, the species tends to colonize substrates presenting different features, with a particular affinity for the deeper zones of the cave, where it probably finds more suitable microclimatic conditions. This broad tolerance reflects the species’ high ecological plasticity, which may also account for the morphological variability observed among individuals from different sampling sites.

The regression analysis revealed a significant relationship between the species’ traits (body and antenna I length) and altitude among the specimens of *B. aetnensis*. Previous studies on beetles have extensively investigated variations in body traits along altitudinal gradients, which have been associated with factors such as predation pressure, food availability, or microhabitat variation (e.g., Baranovská and Knapp. [40], Bhusal et al. [41], Talarico et al. [42]).

Tentatively, this trend may also be attributed to the variability of the volcanic substrate, which generally features wider spaces in younger substrates that are typically more prevalent at higher elevations. The quasi-significance relationship between lava age and body size suggests this trend, although more data on a broader range of lava age conditions would be necessary to fully elucidate this association. A similar pattern was observed in the same region for the subterranean beetle *Duvalius hartigi* Magrini, Baviera & Vigna Taglianti 2006, with the species displaying notable variation in morphological traits due to the diverse environmental conditions within the volcanic substrate [43].

## 5. Conclusions

Our successful documentation of this species was primarily achieved through the careful utilization of pitfall traps, as previous studies relying solely on visual observations proved insufficient in detecting its presence.

In relation to the relative geological youth of the substrates [24,27], the faunal population of Mount Etna depends on colonization processes from the neighboring areas, mainly the Nebrodi and Peloritani Mountains. This process has occurred due to the relatively young age of the volcanic substrates and frequent disturbances caused by volcanic activity.

Consequently, truly paleoendemic elements are missing on Mount Etna, except for those possibly migrated from other neighboring areas. Furthermore, the natural disturbance linked to the volcano’s activity, with emissions of lava, lapilli, ashes, etc., significantly contributing to the rarefaction of the soil fauna of Mount Etna, rendering it notably poorer compared to neighboring areas.

In this particular context, the lava caves, even relatively recent ones, take on significant importance in providing more stable ecological conditions for the soil biocenoses. Within these environments, many species that require varying soil humidity levels can find refuge, and the fallout of ash or lapilli on the ground becomes a critical limiting factor.

The new discovery on Mount Etna highlights the importance of intensifying sampling efforts in the region. Such efforts are crucial, not only for strengthening the protection of these largely unexplored environments, but also for unraveling numerous questions concerning the biogeographic origins of the fauna inhabiting this area. Being a relatively young volcanic environment, the colonization of species has occurred relatively recently, making it an intriguing subject of investigation. For this reason, Mount Etna represents a highly significant natural laboratory to study these processes, the understanding of which is essential for correctly implementing management measures and renaturation projects based on scientific methodologies, rather than relying solely on aesthetic criteria, as unfortunately happens in many protected areas today.

## Figures and Tables

**Table 1:** Occurrences of *Bryaxis aetnensis* sp. nov. on Mount Etna with details of sampling sites. CT is the acronym of the province of Catania.

N.	Locality [Cadastral Number]	Municipality (Province)	Mean T (°C)	Elevation (m a.s.l.)	Age (yrs.)
1	Grotta Lunga (di Monpeloso) [SICT1029]	Nicolosi (CT)	NA	820	1721
2	Grotta di Monte Cicirello [SICT1156]	Trecastagni (CT)	12.62	990	1171
3	Grotta Forcato [SICT1013]	Mascali (CT)	11.23	670	1001
4	Grotta del Porcospino [SICT1033]	Linguaglossa (CT)	11.18	982	1321
5	Grotta del Santo (di San Nicola) [SICT1032]	Adrano (CT)	12.82	1041	>9000
6	Grotta di Monte Corruccio [SICT1056]	Castiglione di Sicilia (CT)	6.49	1389	841
7	Grotta dei Rotoli [SICT1239]	Linguaglossa (CT)	8.53	1488	156

## Data Availability

The data presented in this study are available in this article and in the Supplementary Material.

## Supplementary Materials

The following supporting information can be downloaded at: https://www.mdpi.com/article/10.3390/ani13182941/s1, Table S1: Body traits of *Bryaxis aetnensis* sp. nov. specimens collected on Mount Etna lava caves. Measures of holotype are taken as reference.
