# Supplementary material for: A New Species of Bryaxis (Coleoptera: Staphylinidae: Pselaphinae) from Mount Etna (Sicily, Italy) and Notes on Its Ecology and Distribution
Source: Animals (Basel). 2023 Sep 16;13(18):2941. doi: 10.3390/ani13182941 (PMC10525367; doi:10.3390/ani13182941)
Supplement: Supplementary file 1 [file animals-13-02941-s001.zip › animals-2564611-supplementary.pdf]

Table S1. Body traits of *Bryaxis aetnensis* sp. nov. specimens collected on Mount Etna lava caves. Measures of holotype are taken as reference.

Specimen	Long	Coulor	Eyes omma	Head l/w	Antenna l	Scape l/w	Pedicel l/w	Last palpomere l/w	Pronotum l/w	Elytra l/w	Protibiae	Metatibiae	Aedeagus l
Holotype Grotta dei Rotoli [SICT1239]	1.7	Entirely testaceous reddish	4	0.275/0.29	0.68	0.12/0.06	0.062/0.05	0.285/0.085	0.35/0.35	0.58/0.63	Slightly incised in the distal quarter	Slightly incised in the distal quarter	0.26
Paratype m Grotta di Monte Corruccio [SICT1056]	1.7	Entirely reddish	3	0.275/0.285	0.67	0.11/0.05	0.062/0.045	0.285/0.085	0.33/0.34	0.58/0.625	Slightly incised in the distal quarter	Slightly incised in the distal quarter	0.26
Paratype f Grotta Lunga (di Monpeloso) [SICT1029]	1.45	Entirely testaceous reddish	3	0.265/0.275	0.62	0.11/0.05	0.06/0.045	absent	0.32/0.34	0.56/0.60	-	-	-
Paratype f Grotta del Santo (di San Nicola) [SICT1032]	1.6	Entirely testaceous reddish	4	0.275/0.29	0.635	0.12/0.05	0.062/0.045	0.275/0.08	0.32/0.35	0.56/0.60	-	-	-
Paratype f Grotta di Monte Corruccio [SICT1056]	1.6	Entirely testaceous reddish	4	0.275/0.29	0.64	0.11/0.05	0.06/0.045	0.3//0.085	0.34/0.33	0.55/0.60	-	-	-
Paratype f Grotta del Santo (di San Nicola) [SICT1032]	1.55	Entirely reddish	4	0.265/0.275	0.62	0.11/0.05	0.06/0.045	0.275/0.08	0.34/0.33	0.56/0.60	-	-	-
Paratype f Grotta Lunga (di Monpeloso) [SICT1029]	1.5	Entirely testaceous reddish	3	0.265/0.275	0.62	0.11/0.05	0.06/0.045	0.275/0.08	0.34/0.33	0.56/0.60	-	-	-
Paratype f Grotta di Monte Cicirello [SICT1156]	1.65	Entirely testaceous reddish	3	0.27/0.27	0.65	0.12/0.05	0.06/0.045	0.275/0.08	0.34/0.345	0.58/0.62	-	-	-
Paratype f Grotta Forcato [SICT1013]	1.5	Entirely testaceous reddish	3	0.26/0.27	0.62	0.12/0.05	0.06/0.045	0.275/0.085	0.34/0.34	0.58/0.61	-	-	-
Paratype f Grotta del Porcospino [SICT1033]	1.7	Entirely testaceous reddish	3	0.26/0.27	0.62	0.12/0.05	0.06/0.045	0.28/0.085	0.35/0.35	0.58/0.62	-	-	-
Paratype f Grotta del Porcospino [SICT1033]	1.7	Entirely testaceous reddish	3	0.26/0.27	0.62	0.12/0.05	0.06/0.045	0.285/0.085	0.34/0.34	0.57/0.62	-	-	-
